# Reactivation, Replay, and Preplay: How It Might All Fit Together

**DOI:** 10.1155/2011/203462

**Published:** 2011-09-13

**Authors:** Laure Buhry, Amir H. Azizi, Sen Cheng

**Affiliations:** ^1^Mercator Research Group “Structure of Memory”, Ruhr-University Bochum, Universitaetsstraße 150, 44801 Bochum, Germany; ^2^Faculty of Psychology, Ruhr-University Bochum, Universitaetsstraße 150, 44801 Bochum, Germany; ^3^International Graduate School of Neuroscience, Ruhr-University Bochum, Universitaetsstraße 150, 44801 Bochum, Germany

## Abstract

Sequential activation of neurons that occurs during “offline” states, such as sleep or awake rest, is correlated with neural sequences recorded during preceding exploration phases. This so-called *reactivation*, or *replay*, has been observed in a number of different brain regions such as the striatum, prefrontal cortex, primary visual cortex and, most prominently, the hippocampus. Reactivation largely co-occurs together with hippocampal sharp-waves/ripples, brief high-frequency bursts in the local field potential. Here, we first review the mounting evidence for the hypothesis that reactivation is the neural mechanism for memory consolidation during sleep. We then discuss recent results that suggest that offline sequential activity in the waking state might not be simple repetitions of previously experienced sequences. Some offline sequential activity occurs before animals are exposed to a novel environment for the first time, and some sequences activated offline correspond to trajectories never experienced by the animal. We propose a conceptual framework for the dynamics of offline sequential activity that can parsimoniously describe a broad spectrum of experimental results. These results point to a potentially broader role of offline sequential activity in cognitive functions such as maintenance of spatial representation, learning, or planning.

## 1. Introduction

*Reactivation* of neural activity in the hippocampus was first studied in 1989 by Pavlides and Winson [1]. The authors recorded spiking activity of place cells, hippocampal neurons that are selectively active in restricted regions of space [2]. After determining the so-called place field of a particular neuron, they either allowed the animal to run through the place field (exposure condition) or prevented the access to it (nonexposure condition). As a consequence, the place cell was active in the exposure condition, but inactive in the nonexposure condition. Intriguingly, the activity level of cells during subsequent sleep reflected their earlier activity level during exploration, showing that place cells are reactivated during sleep. Following this finding, investigators first studied pairs [3, 4] and then larger ensembles of place cells [5, 6]. These studies reveal that place cells are activated in a consistent sequential order both during rapid eye movement (REM) sleep [5] and during slow-wave sleep (SWS) [6]. Moreover, the order during sleep matches the order in which the same cells were active during the preceding run on a linear track. This phenomenon is therefore called *replay*. During SWS sleep, reactivation largely co-occurs with hippocampal sharp-waves/ripples (SWR), brief (*≃*80 ms) high-frequency bursts (100–250 Hz) that appear in the local field potential (LFP) in the hippocampus [7–9]. Nevertheless, it remains unclear whether all SWRs are accompanied by replay events and vice versa.

Since the rodent hippocampus is required for learning about new places [10], as well as sequences of nonspatial items [11], it was suggested early on that reactivation might be involved in learning. Specifically, reactivation is thought to be a neural mechanism for consolidation, a process through which memories gradually become independent of the hippocampus [12]. It has been suggested that memories are initially stored in the hippocampus and then gradually transferred to neocortical areas through reactivation in a two-stage process [13, 14].

In this paper, we discuss three classes of experimental findings and their associated functions. First, mounting evidence suggests that reactivation during sleep plays a functional role in consolidation. Second, more recently, reactivation has been observed during the awake state, which differs in intriguing ways from sleep reactivation. Third, recent results suggest that sequential neuronal activation during offline states is not always a replay of sequences previously driven by sensory stimuli [15, 16]. We introduce the term “offline sequential activity (OSA)” to refer to sequential neural activation that is not apparently driven by sensory stimuli. “OSA” is meant to be a catch-all term that potentially includes several types of activity such as replayed sequences, sequences that are not repetition of previously experienced sequences, sequences with a functional relevance, and those without one. We suggest a conceptual framework for the dynamics of OSA that can explain many experimental findings in the three classes of the reviewed phenomena.

## 2. The Link between Reactivation during Sleep and Consolidation

Consolidation can be defined as “a process that transforms new and initially labile memories encoded in the awake state into more stable representations that become integrated into the network of pre-existing long-term memories. Consolidation involves the active re-processing of fresh memories within the neuronal networks that were used for encoding them. It seems to occur most effectively off-line, [*⋯*] so that encoding and consolidation cannot disturb each other and the brain does not hallucinate during consolidation” [17]. This view of consolidation would strongly suggest that it takes place during sleep, as others have suggested before [18, 19]. Here, we review the mounting experimental evidence that reactivation during sleep plays an important role in the process of consolidation.

### 2.1. Memory Performance Is Linked with SWRs and Reactivation

The most direct lines of evidence for a role of reactivation in consolidation are the correlations between memory performance, on the one hand, and SWRs and reactivation, on the other hand. Axmacher et al. [20] presented epilepsy patients with sequences of pictures (landscapes and houses) while recording LFP in the hippocampus and rhinal cortex with standard macro electrodes. The number of successfully recalled items after an *≈*1 h nap is correlated with the number of rhinal, though not hippocampal, SWRs. Dupret et al. [21] showed in rats that the number of reactivation events is correlated with the number of new goal locations that animals can successfully retrieve. These results provide only a correlation between memory performance and reactivation or SWRs, so they do not show that reactivation and/or SWRs are causally driving consolidation.

To show a causal relationship, Girardeau et al. [22] and Ego-Stengel and Wilson [23] interrupted reactivation in rats during sleep. The authors detected the onset of SWRs in the LFP and, upon detection, stimulated the commissural fibers, which bilaterally connect the hippocampi. Such stimulation can suppress CA3 activity, and thus the propagation of replay sequences. Stimulated animals show a small, but significant, memory deficit relative to controls. The residual learning can be explained perhaps by incomplete SWR detection (detection rate approx. 85%), thus allowing some replay events to occur and to potentially drive residual consolidation [22]. It is also possible that some replay events are not accompanied by SWRs and, therefore, cannot be detected by the methods used in these studies. A careful study of the exact relationship between replay events and SWRs has yet to be done.

Even though the deficit in stimulated animals is somewhat small, the results show a clear impairment of memory consolidation due to the suppression of SWRs.

### 2.2. Spiking during Reactivation Can Drive Synaptic Plasticity

We next turn our attention to the mechanism by which reactivation might drive consolidation. The most discussed possibility is that experience of a sequence leads to replay them then drives plasticity, which in turn strengthens the memory of the replayed experience. This chain of arguments can be evaluated at several points. For one, we would expect that more experience (either recent [24, 25] or total experience [3, 13]) leads to more replay. Indeed, sleep replay seems to depend on repeated sequential experiences [25]. The rates of SWRs and reactivation increase with the number of repetitions and the regularity of the behavior in both CA1 and CA3. O'Neill et al. [26] showed that the more time animals spent within the overlap of two place fields, the more the corresponding place cell pair are reactivated during sleep.

Next, we consider whether reactivation could drive synaptic plasticity. Spiking in CA1 pyramidal cells during SWRs is locked to the cycle of the ripple [7], and thus highly similar to tetanic stimuli frequently used to induce long-term potentiation (LTP) in experimental settings [27]. Moreover, spikes fired by neuron pairs in a SWR fall within a time window of tens of milliseconds, consistent with spike-timing-dependent plasticity (STDP) [28–30]. Hence, spiking during reactivation clearly has the properties to drive LTP and STDP.

It has been claimed that replayed sequences are compressed in time by a factor of about 20 relative to the sensory-driven sequences [6, 31]. This apparent compression is, however, an analysis artifact, when spiking during SWRs is compared to the average time it takes the animal to move between the place fields. A better reference are perhaps the firing sequences generated during exploration due to theta phase precession [32]. Phase precession has been observed in individual passes through a cell's place field [33] and, therefore, can generate neural sequences on the order to tens of milliseconds.

### 2.3. Reactivation Occurs in Several Networks throughout the Brain

Since consolidation presumably involves the transfer of information to neocortical areas, reactivation should be observed in brain regions outside of the hippocampus. While most work on reactivation was done in the hippocampus, reactivation was also observed in several other brain structures. First evidence of neocortical reactivation was demonstrated in pairwise correlations during SWS [34]. In the primary visual cortex (V1) and the neighboring secondary visual cortex, Ji and Wilson [35] found that firing sequences evoked by awake experience are replayed during SWS. Similarly to hippocampal place cells, the firing activity of V1 cells is tied to specific locations within the environment; however, spiking of V1 cells is probably driven by local visual, rather than spatial, cues.

Replay was observed in prefrontal cortex (PFC) in transient episodes during SWS [36, 37]. These sequences are compressed in time compared to the average activity during behavior as it is in the hippocampus, but with a factor of 6 to 7 [36]. However, it remains unclear whether a phenomenon similar to phase precession in the hippocampus could explain the apparent compression in prefrontal cortex.

Furthermore, SWR and reactivation were also observed in the ventral striatum during SWS after a task on a track (T maze or triangular track) where rewards were present [38–40]. The striatal reactivation does not seem to decay across 40 min of sleep after the task and is associated with short time intervals after ripple onset [38, 39]. However, in contrast to replay in the hippocampus, this reactivation does not appear during REM sleep and seems to be a sparse phenomenon in the sense that a minority of firing units (coding for the track exploration) are reactivated.

Finally, SWR-like events were recorded in the rhinal cortex of humans by [20], in the entorhinal cortex of rats [41], and in the LFP in cortical sensorimotor and association areas 3, 4, 5, 7, 17, 18, and 21 of cats [42].

### 2.4. SWRs and Reactivation Are Coordinated between Neocortex and Hippocampus

To transfer information from the hippocampus to neocortical areas, activity in different brain regions has to be coordinated. Hippocampal SWRs are presumed to be means for information exchange between the hippocampus and neocortical areas during consolidation [13]. While a coherent picture of these interactions is lacking, many pieces of the puzzle have emerged. For instance, the occurrence of prefrontal sleep spindles and that of SWRs are correlated [43], as are SWR-like events in rhinal cortex and hippocampus [20].

A number of studies have focused on the neocortical up and down states that occur during SWS and in anesthetized animals. Up states are characterized by depolarized membrane potentials and high neural activity and down states by hyperpolarized membrane potentials with little spiking activity [44, 45]. SWRs in the hippocampus apparently appear more frequently during down states, particularly near the transition from the down to the up state [46–48]. A potential coupling mechanism might be the strong synchronization between the membrane potential of hippocampal interneurons and up-down transitions in neocortical LFP [49]; however, up-down states have not been found in the hippocampus itself [35, 50]. Consistent with the hypothesis that different neocortical areas need to be coordinated during consolidation, up-down transitions are correlated across different neocortical sites [51] as well as between cortical areas and hippocampal subareas [50].

The synchronization across brain regions extends to spiking activity as well. Ji and Wilson [35] observed that during SWS there are distinct periods of low and high spiking activity in both hippocampus and V1. The authors called periods of high activity *frames* and suggested that frames correspond largely to cortical up states although they could not be sure due to the lack of membrane potential recordings [35]. The results suggest that frames in the hippocampus and V1 are synchronized as well as replay sequences in the two areas. Frames appear to be involved in the generation of SWRs since hippocampal frames are frequently followed by SWRs about 30 ms later.

Furthermore, hippocampal SWRs are associated with reactivation of neurons in PFC [37, 52] and in ventral striatum [38, 40], which receives direct inputs from CA1 and subiculum [53]. Hoffman and McNaughton [54] studied cell pairs in different neocortical areas of nonhuman primates during a visual task and subsequent sleep. They found that the patterns of correlations during the task phase is reactivated during sleep. Finally, the number of reactivation events is correlated with the density of up-down states [55].

In addition to suggesting correlations between areas, the simple information transfer view implies that information flows from the hippocampus to the other brain regions. Consistent with this expectation, the hippocampus appears to lead the ventral striatum [40] and prefrontal cortex [37]. Some evidence, however, point to a flow of information in the opposite direction. Sirota et al. [46] showed that neuronal bursts in somatosensory cortex trigger SWRs in the hippocampus, Hahn et al. [49] suggested that prefrontal up-down states influence the probability of replay events in the hippocampus, and Ji and Wilson [35] showed that frames in V1 lead the hippocampal frames by 50 ms. These experimental observations suggest that the interactions between hippocampus and other brain regions during consolidation is more complex than the simple view. Interactions might be bidirectional and the direction might change dynamically throughout the consolidation process [37].

### 2.5. Reactivation Occurs in Several Different Species

Since memory consolidation is believed to be a general principle of memory storage, we would expect that a similar mechanism underlies consolidation in different species. Indeed, there is much evidence for SWRs and reactivation in species other than rodents.

Recordings obtained from epileptic patients reveal SWR-like events in the LFP in the human parahippocampus [56], hippocampus, and rhinal cortex [20]. In these studies, SWRs occur only during slow wave sleep or quiet rest with eyes closed and are not observed during the active awake state. Interestingly, single units in the human hippocampus appear to represent spatial locations not unlike place cells in rodents [57]. In nonhuman primates (rhesus macaques), SWRs were observed in the hippocampus and neighboring structures [58]. The authors suggest that SWRs originate mainly in CA1, like they do in rats. Reactivation was observed in the macaque motor, somatosensory, and posterior parietal cortices but not in prefrontal cortex [54]. SWR-like events were detected in the LFP of other mammalian brain regions such as cat cortex [42] and rabbit hippocampus [59].

Finally, replay has been observed in at least one nonmammalian system: area RA of songbirds [60]. The sequential activity of sensorimotor neurons during sleep matches their activity during daytime singing when they drive song production.

## 3. Reactivation during the Awake State and Its Enhancement

Consolidation occurs during sleep, so the popular argument goes, because then the brain is not burdened by stimulus-driven neural activity that could interfere with the network restructuring during consolidation. Similarly, the initial assumption was that reactivation only occurs during sleep [3, 4, 8]. However, Kudrimoti et al. [61] later reported that reactivation occurred in the awake state, too. O'Neill et al. [62] showed that the hippocampal network during the awake state is simultaneously driven by both internal dynamics and sensory inputs. The latter result was found in exploratory SWRs (eSWRs), which the authors defined as SWR that occur during brief periods of rest, that is, within 2.4 s of theta activity, which ceases when the animal remains stationary [62].

### 3.1. Accounting for the Special Properties of Awake Reactivation

During the awake state, SWRs are also frequently accompanied by sequential activation of place cells [24, 63, 64]. However, these sequences exhibit a fascinating difference to reactivation during sleep. Some sequences of activation during quiescent periods are reversed as compared to the sequence during run [24]. It is remarkable that this stunning experimental observation had been predicted by Buzsáki [13] based on a moving-threshold model. In this model, place cells receive subthreshold inputs even when the animal is located far away from its apparent place field. During SWRs, the threshold for spiking is lowered gradually. As the threshold drops, one place cell fires spikes first, the cell that receives the strongest subthreshold excitation, that is, the one with a place field closest to the animal's current location. As the threshold is lowered further, cells with progressively weaker excitation, that is, cells with place fields further and further away, fire spikes, generating a neuronal sequence. When place fields are unidirectional, the moving-threshold model predicts sequences in both the forward and reverse order.

While Foster and Wilson [24] originally reported awake replay in the reverse order, later studies reported awake replay in both the forward and reverse directions on a linear track [64, 65], and in an open environment [63]. When the animal pauses in-between runs along the linear track, forward replay is observed mostly just before a run, suggesting an anticipation of the run [64]. On the other hand, reverse replay is observed just after a run was completed, suggesting a mechanism that uses the information about the outcome of the run to correct the preceding action.

The moving-threshold model made a second important prediction: replay is initiated at the animal's current location. There is indeed a tendency for awake replay to be initiated at or modulated by the animal's current location [62–64]. However, it seems that awake replay can be initiated at remote locations on the track [65] and may correspond to a different track altogether [66]. Furthermore, the fundamental assumption of the moving-threshold model are challenged by recent intracellular recordings [67, 68]. These studies suggest that there is no subthreshold excitatory inputs to place cells distant from their spiking place field.

The experimental evidence on awake replay is not unambiguous. While reactivation is observed in PFC, it apparently does not appear in rest periods that are not classified as SWS [36, 37]. It also remains controversial whether to include SWRs-like events that are observed while the animal is running at significant speeds [62, 69].

What might be the functional role of awake reactivation? This role could be very different from sleep reactivation and possibilities include planning, modifying neural representations, attention, motion, and memory retrieval. Alternatively, awake and sleep replay might serve a similar function: consolidation. If consolidation set in only after the animal had fallen asleep, perhaps hours later, intervening neural activity might overwrite the information about the cells' activity during behavior. This problem could be circumvented by starting the process of consolidation immediately after the experience [70]. If this were the case, we would expect that reactivation was increased by factors that are known to correlate with memory demand or performance. We briefly review the available evidence in the following, and refer the reader to Carr et al. [70] for more detail.

### 3.2. Ripple-Associated Activity Enhanced by Novelty

To study memory formation and/or consolidation we need to examine neural activity when new associations are learned. While some earlier studies found SWR-associated reactivation following exposure to a novel environment [6, 61], they did not compare the neural representations of novel and familiar locations. Foster and Wilson [24] reported that it is easier to observe reactivation of novel linear tracks than of familiar tracks; that is, *P* values indicating the statistical significance of replay were lower after exposures to novel environments than after exposure to familiar environment. This result suggests that there is a difference between the reactivation of novel and of familiar environments, but it does not point to the source of the difference. We, therefore, studied SWR-associated neural activity, while rats alternated between a familiar and a novel arm in an eight-arm maze [69]. Place cells that represent the novel arm show significantly increased activity during SWRs as compared to cells representing the familiar arm. Furthermore, spiking of novel arm cells during SWRs have a higher temporal precision and significantly more SWRs occur when the animal is located in the novel arm. At the same time, spiking activity as a function of spatial location and theta phase is less regular in the novel arm. We, therefore, proposed that enhanced SWR-associated activity drives the formation of precise spatiotemporal representations [69]. Interestingly, in this context, the number of goal-associated eSWRs in a spatial task is correlated with memory performance [21].

Enhanced replay of memory traces during SWS due to learning was also observed in the hippocampus, neocortex, putamen and thalamus [71] although there are methodological concerns with their data analysis [72]. Other studies also reported an increase in SWRs during sleep after exposure to novelty in an association task [73] and a spatial discrimination experiment [74]. Here, learning seems to increase the number of hippocampal SWRs during the first hour of postlearning SWS. Rats that did not learn the discrimination during the training session do not show any change in the number of SWRs.

**Figure 1:**
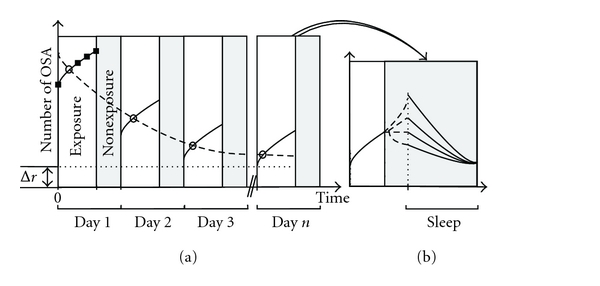
The dynamics of offline sequential activity (OSA) as a function of exposure. This schematic summarizes the results of many reactivation studies in novel and familiar environments. (a) The number of OSA within a day increases with the length of exposure (solid line segments). At the same time, the level of OSA decreases across days as the animal becomes familiar with a novel environment (dashed line). Δ*r* represents the amount of intrinsic OSA (Δ*r* = 0 implies that OSA are purely reactivation of prior sensory-driven sequences). The grey-shaded regions are compressed, and the dynamics within these regions is omitted here for clarity. They are shown in the next panel. (b) Dynamics of OSA within one day. The solid lines are based on experimental observations. The dashed lines represent different hypotheses about the unknown dynamics of OSA between the end of the exposure and the beginning of the sleep phase.

At this point, one might wonder how it is possible that reactivation increases with time spent in the environment, as discussed in the previous section, and decreases with familiarity, as discussed here. We illustrate how both trends can occur at the same time in a schematic representation of the potential dynamics of reactivation (Figure 1). Each solid line segment in Figure 1(a) shows that reactivation within a day increases with the duration of exposure; however, the heights of the solid line segments decrease with each day of exposure before reaching an asymptote. To be consistent with our argument in the next section, we already use the more general term offline sequential activity (OSA), which also includes replay (Figure 1). 

### 3.3. Reactivation Is Enhanced by Reward and Affective State

Reward and affective state are known to influence memory formation [75–78]. We would, therefore, expect that reward and affective state also change reactivation. In most experiments, animals are rewarded for their performance to drive learning. Once the task is learned, the reward is obtained consistently, leaving few unrewarded trials to analyze. By switching the reward contingency mid-session without a signal to the animal, recent experiments were able to induce a period with a significant number of mistakes leading to unrewarded trials [39, 79]. CA3 place cells are more active during SWRs following rewarded trials as compared to unrewarded trials [79]. The enhancement is associated with the reward location: cells with place fields near the reward locations have more enhanced reactivation than others [79]. This enhancement could allow the animal to learn the relationship between the path and the outcome. The goal (or a reward location) is a particularly important location. Indeed, the formation of the goal's spatial representation and its reactivation is correlated with learning [21]. These results are compatible with an earlier observation that place fields gradually shift towards prospective reward locations over multiple trials of a T maze alternation task [80].

As during sleep, reactivation associated with reward during quiet wakefulness is not only observed in the hippocampus, but also in the ventral striatum [39], known to be involved in reward anticipation [81]. The striatal reactivation generally occurs after the hippocampal replay [40].

Together, these observations show that factors known to be correlated with memory performance also influence OSA in different brain regions, underscoring the potential role of awake reactivation in memory consolidation.

## 4. A New Type of Offline Sequential Activity (OSA)?

In the following, we discuss sequential neural activity that occurs during offline states, but that is not necessarily replay of previously experienced sequences. To emphasize this possibility, we use the term “offline sequential activity (OSA)” for internally generated neuronal sequences during offline states, that may or may not be replay of previously sensory-driven sequences.

### 4.1. Offline Sequences That Do Not Seem to Be Reactivation

Gupta et al. [15] investigated the relationship between experience and content of OSA in CA1 cells. They trained rats to run in a two-choice T maze with two return loops. After the second choice, the animals either turned left or right and then completed the loop to returned to the starting location. Animals had to run forward on the left or the right loop or alternate between the two in a block design. At any given stage of the experiment, animals had, therefore, experienced certain parts of the maze more or less recently. Gupta et al. [15] then analyzed the distribution of intervals between experience and replay events. The distribution is inconsistent with replay of the most recent or the accumulated experience, but is consistent with experience-independent replay. To explain the discrepancy with previous studies, the authors suggested that “… the increase in replay with experience seen in these earlier studies [25, 26] may be due to general experience in the environment rather than the experience of particular trajectories.” In other words, the *x*-axis in Figure 1 is “time spent in the test environment”, not the amount of specific experience such as the number of repetitions of a particular trajectory.

A further decoupling between OSA and experienced sequences emerged. Although the animals were mostly prevented from running in the reverse direction, a similar number of forward and reverse replay events was found [15]. Moreover, shortcut sequences were observed during replay that do not correspond to any previously experienced sequence. The animals were prevented from crossing directly between the left and right loops, yet, a significant number of such OSA occurred.

One issue with this study is that animals had experienced the environment in its entirety, even if they had not experienced those particular trajectories that were replayed, thus making it at least possible that the animal had mentally explored all trajectories that were replayed [82]. These objections were overcome by a recent experiment in mice that found *preplay* [16]. CA1 firing sequences recorded during periods of awake rest are correlated with sensory-induced sequences in an environment experienced only later. The OSA were observed both in forward and in reverse order.

A potential interpretation is that preplay emerges from the network structure, that is, sequences preexist in the network structure and are recruited for encoding new memories [16]. This hypothesis is supported by a recent suggestion that the cellular properties of hippocampal cells predict which cells will become active in a novel environment [67]. The Δ*r* in Figure 1 denotes the amount of intrinsic OSA. Δ*r* = 0 would imply that there are no intrinsic OSA, that is, that OSA are always reactivation of prior sensory-driven sequences. The results of Dragoi and Tonegawa [16] indicate a significant nonzero Δ*r*. Figure 1 also illustrates how this preplay, or intrinsic OSA, might have been missed so far, even though several studies have looked at reactivation before and after novel experience [6, 24, 61, 64]. These studies presumed that any correlation between run and presleep are spurious and thus have to be subtracted from the correlation between run and postsleep [72]. For instance, in the exploration of a familiar environment, Kudrimoti et al. [61] found that the pairwise correlations during SWS preceding run are significantly related to those during run. They then proceeded to substract this presleep-run correlation from the postsleep-run correlation for the analysis of reactivation. In addition, correlation-based measures probably lack the statistical power to detect the effect of the relatively small number of significant preplay events [16]. In summary, the most recent results suggest that the level of OSA is not driven by the specific sensory experience and OSA does not correspond to previously experienced sequences.

### 4.2. Possible Functions for Awake Offline Sequential Activity

We next turn to the question what function OSA might serve, when the OSA is not a repetition of previous activity. We previously suggested that SWR-associated activity might drive the formation of spatial representation in novel environments, since SWR-associated activity is enhanced while spatio-temporal spiking is less organized [69]. Consistent with this view, Gupta et al. [15] suggest that the function of forward and reverse replay is to establish and maintain representations of the environment, rather than to replay specific recent experience, since the level of OSA appear to be driven by the familiarity to the environment, and not by the amount of sensory-driven neural activity during behavior. These suggestions are consistent with the idea of maintaining cognitive maps [83, 84].

As preplay occurs before the exploration task, we believe it to be consistent with the notion of planning [65, 85]. Hopfield [82] proposed a model that could support this function. This model, based on the continuous attractor model of Samsonovich and McNaughton [86], includes a hippocampus-like network and allows for mental exploration of trajectories never actually experienced. The goal of this mental exploration could be trajectory planning, that is, finding the optimal path between two locations.

OSA are indeed often observed before choosing a trajectory in spatial tasks or before obtaining a reward; it might, therefore, play a role in predicting the reward location and ingestion [38], possibly through a reverse replay phenomenon [64]. Johnson and Redish [87] suggest that the sequential activity of hippocampal neurons seem to represent future situation rather than recent experience.

Other suggestions focus on the function of OSA in memory and are not explained by the above-cited studies. Dragoi and Tonegawa [16] propose that preplay may facilitate future learning when “a new experience is introduced with multiple steps of increasing novelty”. The presence of OSA could allow for the integration of novel information into a network of older memories. In other words, the sequences would be naturally present in the neural network and would be utilized during a learning experience to store new memory traces.

### 4.3. Sleep Offline Sequential Activity Revisited

The recent results on awake OSA, and the wide range of possible functions that they suggest, call for a reconsideration of sleep OSA. We began our paper with a review of the mounting evidence for the link between reactivation and consolidation. In our view, this link is still the best supported by experimental evidence. However, new possibilities for sleep OSA have opened up and a few nagging question have not been answered so far.

For example, we do not know if sleep OSA is only replaying previous experience. Dragoi and Tonegawa [16] showed preplay during “sleep/rest” periods. We do not know whether preplay occurs during the awake or sleep state or both. Given that many results on awake OSA were only revealed through careful analysis after they were initially overlooked, it seems worthwhile to reexamine sleep OSA in similar ways. Analyses of OSA in both sleep and the awake state in one and the same experiment are needed to determine whether sleep and awake OSA are fundamentally different, aside from the fact that the latter occurs in the reverse order, too. In this vein, the conflicting reports that replay is independent of experience [15] and that replay reflects the amount of previous exposure [25, 26] might be explained by the fact that the first study was conducted in the awake state and the other two in sleep.

While there is much evidence that sleep OSA is involved in consolidation, the link is not exclusive in either direction. On the one hand, sleep OSA in the hippocampus might not be the sole, or even the main, mechanism of consolidation. In fact, the number of rhinal, but not hippocampal, SWRs is correlated with subsequent memory performance in a study conducted in humans [20]. In rats, consolidation is only partially impaired when reactivation in the hippocampus is suppressed by stimulation [22, 23]. In addition to technical explanations for the residual learning discussed above, it is also possible that replay is only one of several mechanisms involved in consolidation. Other potential mechanisms for consolidation include neuronal synchronization [88, 89], and nonsynaptic plasticity phenomena [90, 91].

In the other direction, consolidation might not be the only function of sleep OSA. For example, a computational modeling study of neocortical-hippocampal interaction found that replay might aid the formation of semantic memories and be necessary for the continued maintenance of episodic memories [92]. This result lends support to the multiple memory trace hypothesis that suggests that episodic memories never become fully independent of the hippocampus [93].

Finally, it remains unclear how exactly replay drives consolidation. We do not know, for instance, whether replay strengthens synaptic weights or weakens recently established synaptic connections. Rasch and Born [94] proposed, for instance, that memory traces were transiently destabilized by reactivation to allow for their stabilization and integration into preexisting long-term memories. Similarly, Mehta [95] suggested that reactivation erases memory traces to create a clean slate for future memories. In rat hippocampal slices that are able to spontaneously produce SWRs, Colgin et al. [96] observed that LTP is impaired probably due to the presence of SWRs. These suggestions are consistent with the broader hypothesis that the main purpose of sleep is synaptic downscaling to restore encoding capabilities of the network [97].

## 5. Conclusion

We have reviewed many of the exciting and important findings about reactivation and replay and proposed that they are part of a larger class of phenomena, which we termed OSA. Recent findings suggest that some instances of awake OSA do not repeat sequences that were previously driven by sensory inputs, suggesting that awake OSA is not necessarily a memory trace. We proposed a conceptual framework that parsimoniously accounts for the major known features of the dynamics of OSA. Mounting evidence suggests that sleep reactivation is involved in consolidation although more work is needed to establish the precise mechanisms and to what extend consolidation is driven by OSA versus other mechanisms. By contrast, the functional role of awake OSA and nonreplay OSA are much less clear.

The ubiquity of OSA highlights the ability of biological neural networks to internally generate sequential activity. As such, the mechanism that generates OSA might be related to mechanisms that generate internal dynamics on different timescales and in different neural systems. We name only a few examples here: internally generated sequences at time scales of seconds in the hippocampus [98], free recall of movie sequences [99], working memory maintenance [100], and internal dynamics in sensorimotor learning [101, 102].

We close this paper by pointing out significant gaps in our understanding of OSA dynamics. The schematic shown in Figure 1(a) is only a rough guess. In particular, it is unknown how strongly the network is driven by novelty (dashed curve) versus exposure time in one session (solid line segments), since these two effects have never been studied in the same experiment. Moreover, OSA during the period after exposure, but before sleep sets in, remains unexplored (dashed curves, Figure 1(b)). Activity during this intervening period could simply store the accumulated memory for later consolidation or already be part of the consolidation process. In conclusion, we think that we have only seen the proverbial tip of the iceberg when it comes to OSA. So stay tuned.

## Glossary

Consolidation: Memory process by which memories gradually become independent of the hippocampus
Frames: Periods of high activity in V1 and the hippocampus believed to correspond largely to cortical up states [35]
eSWR: Exploratory sharp wave/ripple. Occurs during brief periods of rest, within 2.4 s of theta activity [62]
Exposure: Occurs when the animal is placed in an environment and is allowed to explore the environment
Offline state: Behavioral state during which the animal is asleep or resting quietly
OSA (offline sequential activity): Sequential activation of neurons during offline states. A catch-all term that includes activity with or without a functional relevance
Reactivation: Offline activation of neurons that were active together during earlier exploration
REM: Sleep stage characterized by rapid eye movements
Replay: Sequential activation of (>2) neurons during offline states in the same order as previously observed during active exploration
SWR: Sharp wave/ripples. Sharp waves: large deflections in the unfiltered LFP; ripples: brief (*≃*80 ms) high-frequency (100–250 Hz) bursts in the LFP
SWS: Slow wave sleep. Sleep stage characterized by slow oscillations in the EEG.
